# Effect of Continuous Ingestion of Bifidobacteria and Inulin on Reducing Body Fat: A Randomized, Double-Blind, Placebo-Controlled, Parallel-Group Comparison Study

**DOI:** 10.3390/nu15245025

**Published:** 2023-12-07

**Authors:** Yuhei Baba, Yasuo Saito, Mei Kadowaki, Naoki Azuma, Daisuke Tsuge

**Affiliations:** 1 Dairy Business Division, Ezaki Glico Co., Ltd., 4-6-5 Utajima, Nishiyodogawa-Ku, Osaka 555-8502, Japan; 2 R&D Laboratory, Ezaki Glico Co., Ltd., 4-6-5 Utajima, Nishiyodogawa-Ku, Osaka 555-8502, Japan; yasuo.saito@glico.com (Y.S.); mei.kadowaki@glico.com (M.K.); naoki.azuma@glico.com (N.A.); 3 Shinagawa Season Terrace Health Care Clinic, Shinagawa Season Terrace (5F), 1-2-70 Konan, Minato-Ku, Tokyo 108-0075, Japan; shibaura@sempos.or.jp

**Keywords:** *Bifidobacterium animalis* subsp. *lactis*, inulin, synbiotics, abdominal fat, overweight, gut microbacteria

## Abstract

*Bifidobacterium animalis* subsp. *lactis* GCL2505 has been shown to have several positive health effects, including improved defecation frequency and reduced visceral fat. It is known that combined intake of GCL2505 and inulin increases the total number of bifidobacteria compared with ingestion of GCL2505 alone. This randomized, double-blind, placebo-controlled, parallel-group study was conducted to confirm that consumption of GCL2505 and inulin reduces abdominal fat (*n* = 120). Participants consumed a test beverage containing 1 × 10^10^ colony-forming units of GCL2505 per 100 g and 2.0 g of inulin per 100 g for 12 weeks. A change in the visceral fat area (VFA) was set as the primary endpoint. There were significant reductions in VFA and total fat area. The intervention significantly increased the total number of bifidobacteria and affected the levels of several lipid markers. Regression analysis of bifidobacteria and measured parameters showed that total bifidobacteria correlated with VFA and body mass index (BMI), while endogenous bifidobacteria and *Bifidobacterium animalis* subsp. *lactis* correlated only with BMI, suggesting that increases in both contributed to the decrease in VFA. These results suggest that combined intake of GCL2505 and inulin improves the intestinal environment and reduces abdominal fat in association with the SCFA-mediated pathway.

## 1. Introduction

The World Health Organization defines overweight and obesity as abnormal or excessive fat accumulation that has the potential to negatively impact health. Obesity is a risk factor for lifestyle diseases such as hypertension, dyslipidemia and diabetes, as well as noncommunicable diseases such as cardiovascular diseases (mainly heart disease and stroke), musculoskeletal diseases (especially osteoarthritis) and some cancers. The worldwide prevalence of obesity nearly tripled between 1975 and 2016, with more than 650 million adults becoming obese. Overweight and obesity are linked to more deaths worldwide compared with underweight [1]. Thus, the obesity epidemic is one of the greatest public health challenges of the twenty-first century. Abdominal visceral fat accumulation (abdominal obesity) is a form of obesity related to environmental factors such as diet and physical inactivity and is also an underlying component of metabolic syndrome, which is a risk factor for coronary heart disease, hypertension, type-2 diabetes and impaired glucose tolerance. Furthermore, accumulation of abdominal visceral fat is considered to have greater negative health implications compared with obesity in general [2]. Caloric restriction and exercise are commonly used to treat obesity. For severe obesity, bariatric surgery and pharmacotherapy may also be used, but these have issues such as invasiveness and continuity. Therefore, there is a need for treatments such as functional foods, which do not require major lifestyle changes and are easier to apply to daily life [3].

The fundamental cause of overweight and obesity is an energy imbalance between calories ingested and calories expended [1]. In addition, it has been reported that the gut microbiota and obesity are closely related, with the composition and diversity of gut microbiota altered in overweight and diabetes [4]. Accordingly, probiotics and prebiotics may offer one approach to treating overweight and obesity by regulating the gut microbiota. Probiotics are defined as “live microorganisms that, when administered in adequate amounts, confer a health benefit on the host” [5]. Lactic acid bacteria (especially *Lactobacillus* spp.) and bifidobacteria have been reported as probiotics in the treatment of obesity. Consumption of *Lactobacillus gasseri* SBT2055 has been shown to reduce body weight, body fat percentage, body fat mass, visceral fat mass, subcutaneous fat mass, waist circumference, hip circumference, waist-to-hip ratio (WHR) and triglycerides (TG) [6,7], while consumption of strain BNR17 has been shown to reduce visceral fat mass [8]. Consumption of *Lactiplantibacillus plantarum* DAD-13 has been shown to reduce body weight and body mass index (BMI) [9], while strain K50 has been shown to reduce total cholesterol (TC) and TG [10], and strain LMT-1-40 has been shown to reduce body fat mass and abdominal VFA and to change insulin-related parameters [11]. In addition, it has been reported that consumption of *Bifidobacterium breve* B-3 decreases body fat mass, body fat percentage and abdominal VFA [12]. Consumption of *Bifidobacterium animalis* subsp. *lactis* CECT8145 decreases BMI, waist circumference and waist circumference/height [13], while consumption of strain GCL 2505 decreases visceral fat [14]. Furthermore, several meta-analyses have suggested an association of probiotics with glucose metabolism, lipid metabolism, body fat mass, body weight, visceral adipose tissue and hepatic adiposity [15,16,17]. Meanwhile, prebiotics are nonviable food components that confer a health benefit on the host and are associated with modulation of the gut microbiota [18]. Multiple meta-analyses [19,20,21] have shown that prebiotics reduce body weight, BMI, body fat, fasting glucose, insulin and fasting TG. However, the efficacy of a single probiotic depends on various factors, including diet and indigenous bacteria [22,23,24]. In addition, the effect of prebiotics is influenced by the gut microbiota of the individual and its sugar capitalization [24,25]. For these reasons, there may be limitations to using single probiotics or prebiotics to treat obesity in diverse populations. The combination of probiotics and prebiotics is called synbiotics [26], and it has been reported that intake of synbiotics increases gut bifidobacteria [27,28]; even in subjects with low probiotic strain growth, total bifidobacteria counts increased due to an increase in endogenous bifidobacteria [29]. Furthermore, several animal studies have reported that intake of synbiotics acts synergistically against obesity [30,31]. Clinical trials investigating the health benefits of synbiotics in a variety of areas found the following benefits. For intestinal health, there was a reduction in abdominal pain frequency [32] as well as an overall improvement in symptoms of irritable bowel syndrome [33]. For obesity and metabolic diseases, reductions in body weight, BMI [34] and body fat percentage [35] as well as improvements in inflammatory markers [36] have been reported. Improvements in the stress response [37] as well as reductions in tension and drowsiness [38] have also been reported. Therefore, it may be beneficial to consider a synbiotic or multi-strain probiotic approach with a view toward providing benefits to a broader population.

*Bifidobacterium animalis* subsp. *lactis*, a probiotic strain, is commonly used in fermented dairy products and has shown numerous health benefits related to gastrointestinal and immune health [39,40]. *Bifidobacterium animalis* subsp. *lactis* GCL2505 is a probiotic strain isolated from the feces of healthy adults that can grow in the gut [41,42] and it is used in fermented milk products in Japan. In clinical trials, daily consumption of fermented milk containing 8 × 10^10^ colony-forming units (CFU) of GCL2505, which contains higher numbers of bifidobacteria compared with regular fermented milk, reduced abdominal VFA [14]. Also, combined intake of GCL2505 and inulin was shown to be more effective in increasing the total number of bifidobacteria compared with ingestion of GCL2505 alone [29]. Based on these findings, this study evaluated the effects of the synbiotic intake of GCL2505 and inulin on abdominal fat accumulation in overweight Japanese adults in a placebo-controlled, randomized, double-blind, parallel-group study.

## 2. Materials and Methods

### 2.1. Participants

Participants were Japanese men and women between the ages of 20 and 65 years at the time of consent, who satisfied the inclusion criteria, did not satisfy any of the exclusion criteria and were deemed eligible to participate by the principal investigator. The inclusion criteria were as follows: (1) BMI of 23 or higher and less than 30 at the screening test; (2) able to abstain from alcohol for 2 days before each measurement; and (3) fully informed of the purpose and content of the study, deemed to have the capacity to consent, volunteered of their own accord to participate in the study based on a thorough understanding of the purpose and content of the study and provided written informed consent to participate in the study. Exclusion criteria were as follows: (1) regularly taking medications that affect obesity, hyperlipidemia or lipid metabolism; (2) treatment for severe kidney disease, heart disease, respiratory disease, endocrine disease, diabetes or other illness (excluding transient illnesses such as colds); (3) unable to stop intake of health foods or supplements that affect obesity, hyperlipidemia or lipid metabolism; (4) unable to restrict the intake of foods that might affect the intestinal microbiota; (5) use of antibiotics within 1 month prior to the start of the study; (6) regularly use intestinal drugs and laxatives (including strong laxatives); (7) history of digestive surgery (excluding appendectomy); (8) history of allergy to any of the study food ingredients; (9) current or former drug or alcohol dependence; (10) presence of metal (e.g., surgical implants) that precludes computed tomography (CT) scans of the measurement site; (11) implanted medical devices such as cardiac pacemakers and cardioverter-defibrillators; (12) claustrophobia; (13) pregnant or lactating, or expecting to become pregnant during the study; (14) participation in research involving the ingestion of other foods or the use of pharmaceuticals, the application of cosmetics or pharmaceuticals or participation in other research while participating in this study; and (15) deemed ineligible by the principal investigator.

### 2.2. Test Foods

The test products were a dairy drink (active drink) containing inulin (Orafti GR; BENEO GmbH, Mannheim, Germany) and GCL2505 or placebo. The active drink contained 1 × 10^10^ CFU of GCL2505 and 2.0 g of inulin per 100 g. The placebo was prepared using the same ingredients as the active drink, with the addition of food-grade acetic acid and lactic acid to adjust the flavor and pH; the basic ingredients were skim milk powder, fructose, dextrose, sucrose, yeast extract, acidifier, stabilizer and flavoring. The nutritional details of the test products are shown in Table 1.

### 2.3. Experimental Design

This was a randomized, placebo-controlled, double-blind, parallel-group study. Participants were randomized by computer-generated randomization into two groups (1:1), with age at screening, sex, body weight, body fat rate and VFA serving as stratification factors for randomization in block sizes of four. The controller (allocation manager) assigned the two groups to the test drink intake group and the control food intake group. For the sample size, the final target number of subjects was set at 60, referring to previous reports on visceral fat reduction with probiotics [14,43,44]. Participants in the active and placebo groups consumed 100 g of dairy beverage once daily for 12 weeks. Both the participants and observers were blinded to the group allocation for the duration of the study. Double blinding was accomplished by labeling the test drink with only an identification number. The change in VFA between weeks 0 and 12 was set as the primary endpoint. The secondary endpoints were VFA between weeks 0 and 8, subcutaneous fat area (SFA) between weeks 0, 8 and 12, total fat area (TFA) between weeks 0, 8 and 12, body weight, BMI, body fat rate, waist circumference, hip circumference, WHR, TC, low-density lipoprotein cholesterol (LDL-C), HDL-C, triglyceride, free fatty acid and fecal bifidobacteria. The study was conducted at the Shinagawa Season Terrace Health Care Clinic (Tokyo, Japan) from October 2022 to April 2023 by K.S.O. Corporation (Tokyo, Japan), a contract research organization, and was registered with the University Hospital Medical Information Network Clinical Trials Registry (UMIN-CTR) “http://www.umin.ac.jp/ctr/index.htm (accessed on 26 October 2022)” as UMIN000049328. This article conforms to the Consolidated Standards of Reporting Trials (CONSORT) 2010 guidelines (Supplementary Materials, Table S1).

### 2.4. Abdominal Fat Area

Abdominal VFA and SFA were measured using CT. Four-slice CT images (120 kVp, 400 mAs tube current, 5.0 mm slice thickness and 420 mm field of view) were acquired at the level of the lumbar 4 vertebra. Abdominal VFA, SFA and TFA (i.e., visceral + subcutaneous) were measured using Fat Scan ver. 4 (East Japan Institute of Technology Co., Ltd., Hitachi, Japan). To avoid unnecessary radiation exposure, CT scans were conducted only once at each measurement point (0, 8 and 12 weeks). The measurement of VFA by CT is reported to be easily affected by the slice site as well as the respiration phase of the subject [45]. Therefore, to investigate the time course changes in VFA accurately, the scanner and principal investigator strictly assessed a series of CT images obtained from the same subjects at each measurement point, treating any inappropriate data as missing values.

### 2.5. Anthropometric Measures and Body Composition

Weight and height were measured to the nearest 0.1 kg and 0.1 cm, respectively, with the participant standing. BMI was calculated in the standard way: weight (kg) divided by the square of height (m). Waist and hip circumferences were measured to the nearest 0.1 cm in a standing position. Waist circumference was measured around the abdomen at the level of the umbilicus. Hip circumference was measured at the level of maximum extension of the buttocks posteriorly in a horizontal plane.

### 2.6. Clinical Parameters

Blood pressure, pulse rate and the concentrations of biochemical and hematological parameters in plasma were measured at weeks 0, 4, 8 and 12. The concentrations of urinary parameters were also measured at weeks 0 and 12. Blood samples were drawn from each participant after 10 h of no food or drink except water (fasting) prior to testing. Clinical parameters included hematological tests (white blood cell count, red blood cell count, hemoglobin, hematocrit, platelet count and leukogram), biochemical tests (total protein, albumin, total bilirubin, aspartate aminotransferase, alanine aminotransferase, lactate dehydrogenase, alkaline phosphatase, gamma-glutamyltransferase, urea nitrogen, creatinine, uric acid, sodium, chlorine, potassium, calcium, TC, LDL-C, HDL-C, TG, glucose and HbA1c [NGSP]) and urinalysis (protein, sugar, bilirubin, urinary ketone bodies, occult blood reaction, urobilinogen, pH and specific gravity). All of these tests were performed at LSI Medience Corporation (Tokyo, Japan).

### 2.7. Fecal Samples

Fecal samples were submitted at weeks 0, 8 and 12. Fecal samples were collected at home between 7 and 2 days before the specified visit. The submitted samples were promptly transported to the Kyoto Institute of Nutrition and Pathology (Kyoto, Japan) by refrigerated transport at temperatures below −15°C.

### 2.8. Fecal DNA Extraction

Bacterial DNA was extracted from fecal samples, using the ISOSPIN Fecal DNA Kit (Nippon Gene Co., Ltd., Tokyo, Japan), following the procedure of Tourlousse et al. [46]. Specifically, a 0.2 g fecal sample, 700 μL of FE1 buffer and 10 μL of RNase were added to a tube with attached beads. A bead-beating homogenizer (FastPrep-24; MP Biomedicals, Irvine, CA, USA) was used at a rate of 6 m/s for 1 min to crush the cells. The process was repeated three times, during which the sample was kept at room temperature for 5 min. Then, 90 µL of FE2 buffer was added and the samples were centrifuged at 12,000× *g* for 15 min. The supernatant (up to 500 µL) was collected and mixed with FB buffer and isopropanol, each at 0.4× the volume of the supernatant obtained. Finally, the sample was loaded onto a spin column and washed according to the manufacturer’s instructions. Purified DNA was eluted with 50 µL of Tris-EDTA buffer (pH 8.0).

### 2.9. Fecal Bifidobacteria

Bacterial DNA was extracted from 10-fold dilutions of the fecal samples, and the number of gut bifidobacteria was determined by quantitative real-time PCR using bifidobacteria species- and subspecies-specific primers according to a procedure described previously [47]. Total counts of bifidobacteria in the fecal samples are represented as the sum of 10 species (*B. longum* subsp. *longum*, *B. adolescentis*, *B. catenulatum*, *B. pseudocatenulatum*, *B. breve*, *B. bifidum*, *B. longum* subsp. *infantis*, *B. dentium*, *B. angulatum* and *B. animalis* subsp. *lactis*). Endogenous bifidobacteria were regarded as the sum of nine species, without *B. animalis* subsp. *lactis*. The detection limit of each species or subspecies was 2.0 × 10^5^ cells per gram of feces.

### 2.10. Statistical Analysis

All measurements are expressed as mean, standard deviation (SD) and standard error (SE). Statistical analyses were performed using IBM^®^ SPSS^®^ Statistics 27 (IBM Corp., Armonk, NY, USA) or R^®^ 4.2.1 (R Foundation for Statistical Computing, Vienna, Austria). A *p*-value < 0.05 was used as the threshold for determining significance. As basic statistics, means, SDs and SEs are expressed to the nearest significant digit and percentages are expressed to one decimal place, with digits adjusted by rounding. Missing data were treated as missing values and no surrogate values were used. Statistical analysis of VFA, SFA, TFA and fecal bifidobacterium was performed with unpaired *t*-tests, using the Benjamini–Hochberg procedure in order to compare between the active and placebo groups at each examination time. In addition, statistical analyses were performed with paired *t*-tests, using the Benjamini–Hochberg procedure in order to compare the test results at the start of intake (week 0) with those at 8 and 12 weeks after intake. Fecal bifidobacteria counts were converted to ordinary logarithms before performing statistical analysis. For VFA, SFA and TFA, an intergroup comparison was performed using two-factor repeated-measures analysis of variance (ANOVA) with the actual values. For the other items, comparisons between the active and placebo groups at each examination time were statistically analyzed with an unpaired *t*-test (two-tailed). In addition, statistical analysis was performed with paired *t*-tests to compare the test results at the start of the intake with those at 4, 8 and 12 weeks after intake. Regression analysis to correlate bifidobacteria counts with body composition parameters and biomarkers of obesity were performed by applying ANOVA to a mixed linear model, with bifidobacteria count as the objective variable, sex, age, BMI, VFA, SFA, TG, TC, LDL-C, HDL-C, treatment group (active or placebo) and time point (0, 8 and 12 weeks) as explanatory variables and participant ID as a random variable. The lmer function of the R package lmerTest, version 3.1-3, was used for these analyses.

## 3. Results

### 3.1. Analysis of the Participant Population

**Figure 1:**
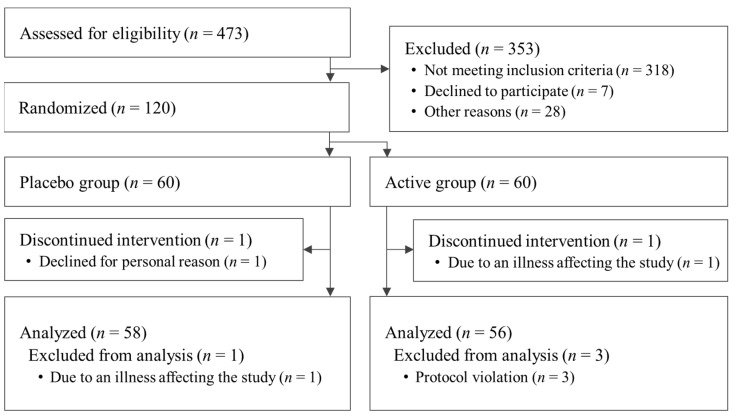
Flowchart of participant selection.

The flow chart of study participation is shown in Figure 1. A total of 473 participants were screened for this study. After screening, 120 participants were eligible: 60 were assigned to the active group and 60 were assigned to the placebo. A significant difference in basophil ratio between the active and placebo groups was observed at the beginning of the study but was deemed acceptable because it was within the reference range. For the other items, there were no differences in the baseline characteristics of the participants’ data (Table 2). Dietary consumption for the three days prior to the measurement is summarized in Table 3. It was concluded that the results of dietary consumption did not significantly affect the results of this study. The fat intake of the active group at week 12 was significantly less than that of the placebo group. The difference in mean fat intake between the active and placebo groups was 8.2 g/day or 73.8 kcal/day in terms of calories. Considering the energy intake recommended for the participants in this study [48], this change was only 2.8–3.8% of the daily energy intake. In addition, the difference between groups in the degree of change in VFA, discussed below, was confirmed from week 8; it was determined that this difference between groups, confirmed at week 12, did not have a significant impact on the study. Also, energy and protein intakes at week 12 were significantly reduced compared with week 0 in both the active and placebo groups. Changes in energy and protein intake from baseline in the active and placebo groups were determined not to have affected the study results because there were no significant differences between the groups. Carbohydrate and fiber intakes at week 12 in the placebo group were significantly reduced compared with baseline but this was determined not to have affected the study results because there were no significant differences between groups. By the end of the study, one participant from the active group withdrew due to an illness unrelated to the study that may have affected the results, and one participant from the placebo group withdrew for personal reasons. After the completion of the entire study, one participant from the placebo group was excluded due to a confirmed illness unrelated to the study that may have affected the results. In addition, three participants were excluded because they were found to have consumed drugs or foods during the study period that might have affected the results (*n* = 1 from the active group and *n* = 2 from the placebo group). Thus, a total of 114 patients (58 in the active group and 56 in the placebo group) were included in the analysis. There were no reported harms or unintended effects in either group.

### 3.2. Abdominal Fat Area

**Figure 2:**
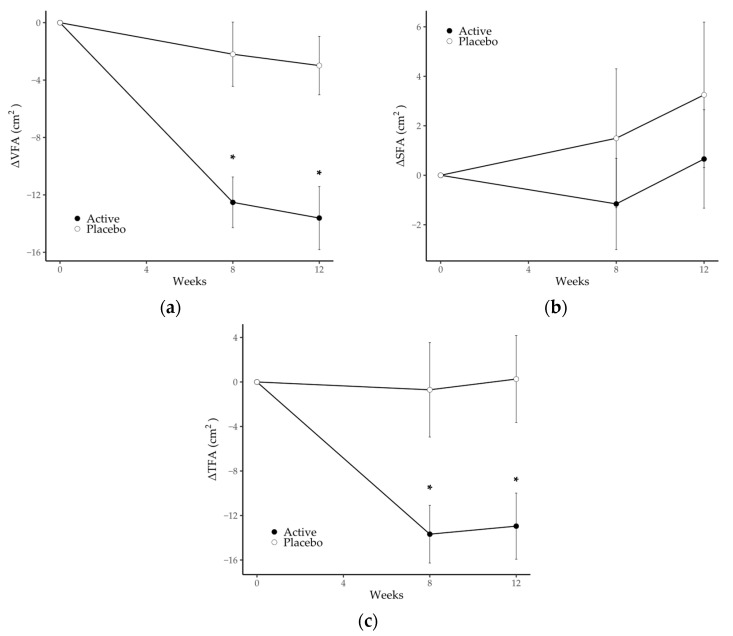
Changes in (**a**) visceral fat area, (**b**) subcutaneous fat area and (**c**) total fat area in the placebo and active groups during the study period. Values are the means, with error bars as standard error. Asterisks (*) indicate a *p*-value < 0.05 as a result of inter-group difference (the placebo group vs. the active group; unpaired *t*-test with Benjamini–Hochberg procedure). SFA, subcutaneous fat area; TFA, total fat area; VFA, visceral fat area.

From the viewpoint of the accuracy of the CT data described in Section 2.4, we assessed a series of CT images obtained from the same participants at each measurement point, treating any inappropriate data as missing values. Data from 16 participants (1 in the active group and 15 in the placebo group) were treated as missing values in part or in whole due to overestimation of VFA, caused mainly by compression of the abdominal cavity during inspiration, and 6 participants (all in the active group) were treated as missing values in part or in whole due to underestimation of VFA, caused mainly by the inclusion of an internal organ or gas in the CT scan images. Consequently, data from 102 participants at week 0 (55 in the active group and 47 in the placebo group), data from 100 participants at week 8 (54 in the active group and 46 in the placebo group) and data from 94 participants at week 12 (52 in the active group and 42 in the placebo group) were analyzed. The mean decreases in VFA from baseline to 8 and 12 weeks, respectively, were significantly greater in the active group (−12.5 ± 1.8 cm^2^ and −13.6 ± 2.2 cm^2^) compared with the placebo group (−3.0 ± 2.0 cm^2^ and −2.2 ± 2.2 cm^2^). In addition, the mean reduction in TFA from baseline to 8 and 12 weeks, respectively, was significantly greater in the active group (−13.7 ± 2.6 cm^2^ and −13.0 ± 3.0 cm^2^) compared with the placebo group (−0.7 ± 4.2 cm^2^ and 0.3 ± 3.9 cm^2^). There were no statistically significant differences in SFA between the two groups and no changes within either group (Figure 2). The actual values of VFA, SFA and TFA are summarized in Table 4; VFA and TFA in the active group at weeks 8 and 12 were significantly reduced compared with baseline. There was a significant group-by-time interaction in VFA and TFA from baseline.

### 3.3. Anthropometric Parameters

Body weight, BMI and WHR values are summarized in Table 5; there were no statistically significant differences in body weight, BMI or WHR between the two groups. Body weight and BMI in the active group at week 12 were significantly lower compared with baseline. The values for waist and hip circumference that were used to calculate WHR are presented in Table S2 of the Supplementary Materials.

### 3.4. Biochemical Parameters in Plasma

The values of TC, LDL-C, HDL-C, TG and free fatty acids in plasma are summarized in Table 6. TG in the active group at weeks 4 and 8 was significantly lower compared with the placebo group. TC, LDL-C and HDL-C in the active group at week 8 were significantly lower compared with baseline. Free fatty acids in the active and placebo groups at week 12 were significantly increased compared with baseline.

### 3.5. Fecal Bifidobacteria

**Figure 3:**
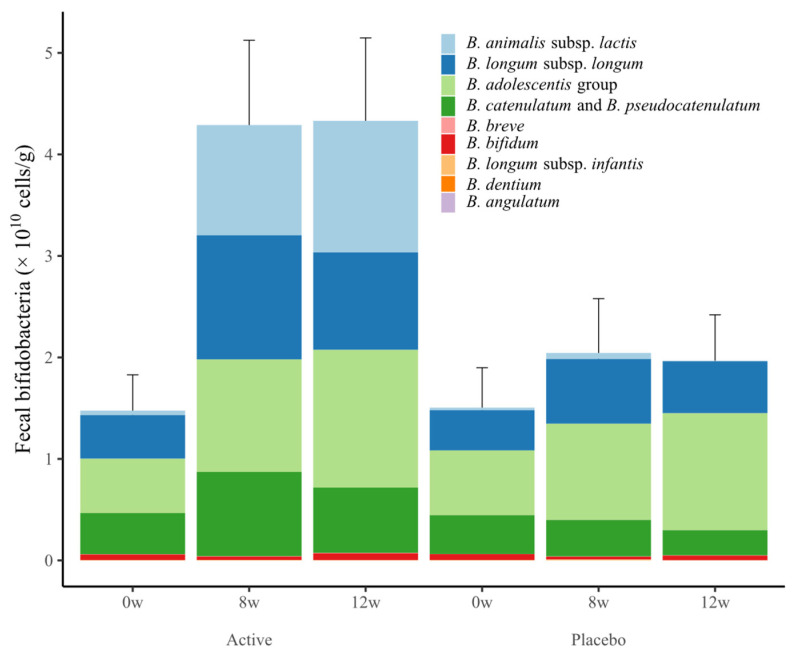
Changes in the number of fecal bifidobacteria in the placebo and active groups during the study period. Values are expressed as the sum of the mean ± standard error values of each species.

Changes in the number of fecal bifidobacteria are shown in Figure 3 and Table 7. Total bifidobacteria, *B. animalis* subsp. *lactis*, *B. catenulatum* and *B. pseudovatenulatum* counts in the active group at weeks 8 and 12 were significantly higher compared with the placebo group. In addition, total bifidobacteria, *B. animalis* subsp. *lactis*, *B. longum* subsp. *longum*, *B. adolescentis* group, *B. catenulatum* and *B. pseudovatenulatum* in the active group at 8 and 12 weeks were significantly increased compared with baseline.

### 3.6. Regression Analysis with Fecal Bifidobacteria and Measured Parameters

Table 8 shows the results of the regression analysis performed to examine the association of total bifidobacteria, endogenous bifidobacteria and *B. animalis* subsp. *lactis* bacteria counts with the participants’ characteristics, body compositions and plasma parameters. Regression analysis showed that an increase in the number of total bifidobacteria was significantly associated with a decrease in BMI and VFA, while an increase in the number of *B. animalis* subsp. *lactis* and endogenous bifidobacteria was significantly associated with a decrease in BMI.

## 4. Discussion

We investigated the effects of consuming a dairy drink containing a synbiotic comprising *Bifidobacterium animalis* subsp. *lactis* GCL2505 and inulin on abdominal fat in overweight adults. The results showed that the consumption of the test beverage resulted in a reduction in abdominal visceral fat and total abdominal fat. In abdominal adipose tissue, visceral and subcutaneous adipose tissue have very different effects on metabolic disorders [49], and several studies have reported that excess VFA, rather than SFA, body weight or BMI, is correlated with metabolic disorders [45,50]. Therefore, abdominal VFA was set as the primary endpoint in this study.

After 12 weeks of consuming a dairy beverage containing GCL2505 and inulin, the reduction in VFA from week 0 to weeks 8 and 12 in the active group was significantly greater than that in the placebo group. Although there was no significant group difference in the reduction in SFA, the reduction in TFA was significantly greater compared with the placebo group, thus confirming the reduction in overall abdominal fat due to ingestion of the synbiotic. In addition, quantification of fecal bifidobacteria showed that total bifidobacteria, *B. animalis* subsp. *lactis*, *B. catenulatum* and *B. pseudocatenulatum* in the active group at weeks 8 and 12 were significantly greater compared with the placebo group. This confirmed an increase in endogenous bifidobacteria due to inulin, as well as an increase in *B. animalis* subsp. *lactis* due to ingestion of GCL2505. In the active group, the total number of endogenous bifidobacteria, *B. longum* subsp. *longum* and *B. adolescentis* was significantly increased at weeks 8 and 12 compared with baseline. These results are in line with a previous study showing that intake of GCL2505 and inulin increases endogenous bifidobacteria (especially *B. longum* subsp. *longum* and *B. adolescentis*) as well as total bifidobacteria counts more compared with GCL2505 alone [29].

At weeks 4 and 8, TG in the active group was significantly lower compared with the placebo group, suggesting an effect of GCL2505 and inulin intake on lipid parameters in plasma. In addition, body weight and BMI at week 12 as well as TC, LDL-C and HDL-C at week 8 in the active group were significantly lower compared with baseline. Changes in body weight and BMI were also reported in a meta-analysis by Koutnikova et al. [17], along with changes in VFA, which showed significant differences between groups. In addition, changes in body weight, BMI, TC and LDL-C were reported in a meta-analysis on synbiotics by Musazadeh et al. [51], along with changes in TG, which showed significant differences between groups. Although further studies are needed because of the absence of differences between groups, it is possible that the changes in these parameters were due to the intake of GCL2505 and inulin. However, the differences between groups in TG at week 12 as well as the differences in TC, LDL-C and HDL-C at week 12 compared with baseline were not significant. In addition, there was a significant increase in free fatty acids at week 12 compared to baseline in both groups. These results might be associated with an increase in the mean changes in TFA from baseline in both groups from weeks 8 to 12. It is hypothesized that intake of GCL2505 and inulin reduced visceral and body fat through a mechanism involving two steps. In the first step, intake of GCL2505 and inulin increases bifidobacteria and production of short-chain fatty acids (SCFAs) in the gut. In this study, total bifidobacteria counts increased significantly in the active group compared with the placebo group. In animal studies, the intake of GCL2505 alone contributed to an increase in the number of fecal bifidobacteria along with a corresponding increase in the concentration of acetic acid in feces and blood [52,53]. In clinical studies, intake of GCL2505 and inulin increased total bifidobacteria counts in feces [29], while intake of inulin alone increased SCFAs such as acetic acid [54] by increasing the number of bifidobacteria in the gut. Thus, in the present study, it was suggested that GCL2505 and inulin in the gut increased acetic acid, one of the SCFAs, by increasing the number of total bifidobacteria. In the second step, the increase in SCFAs in the gut improved glucose tolerance and systemic fatty acid oxidation through their receptor, G protein-coupled receptor 43 (GPR43), leading to a reduction in visceral and body fat. Previous studies in animals showed that increased production of acetic acid improves glucose tolerance, promotes systemic fatty acid oxidation and suppresses body fat accumulation via GPR43 [53]. In addition, intake of inulin led to a reduction in VFA [55]. In clinical studies, it was shown that daily consumption of a test beverage containing a higher amount of GCL2505 (8 × 10^10^ CFU) compared with standard fermented milk reduced abdominal VFA [14]. Taken together, the findings suggest that combined intake of GCL2505 and inulin may increase the concentration of SCFAs in the gut by increasing the total bifidobacteria count, thereby reducing visceral fat and body fat via GPR43.

The differences in TG between groups at week 8 may be due to the action of SCFAs. It was suggested that SCFAs promote lipid clearance in the liver by downregulating angiopoietin-like protein 4, which inhibits lipoprotein lipase [56]. In fact, the reduction in TG associated with *Lactiplantibacillus plantarum* consumption [10] is thought to be due to SCFA-mediated mechanisms of action. In the present study, VFA at baseline in the active group (124.0 ± 31.3 cm^2^) was lower than that reported in a previous study [14] in which GCL2505 alone was ingested eight times (133.4 ± 29.6 cm^2^) and the participants had low visceral fat from the start. Nevertheless, the change in VFA (−13.6 ± 2.2 cm^2^) was greater than that reported in previous trials (−5.1 ± 1.8 cm^2^). In addition, the degree of change was greater than that reported in a meta-analysis [17] (−6.30 cm^2^, 95% CI −9.05, −3.56). In addition, as noted above, combined intake of GCL2505 and inulin was shown in clinical studies to increase the total number of intestinal bifidobacteria more compared with consumption of GCL2505 alone [29]. It is possible that the combined intake of GCL2505 and inulin might have reduced VFA and TFA more effectively in the present study by increasing total bifidobacteria.

The relationship between parameters such as VFA and TG (which changed in this study) as well as the number of bifidobacteria were estimated by regression analysis and applying analysis of variance to a mixed effects model. The results suggest that an increase in total bifidobacteria count is significantly associated with a decrease in BMI and VFA, whereas an increase in *B. animalis* subsp. *lactis* and endogenous bifidobacteria is associated only with a decrease in BMI and not with a decrease in VFA. Because the increase in *B. animalis* subsp. *lactis* and endogenous bifidobacteria does not correlate with the decrease in VFA, it is possible that increases in both *B. animalis* subsp. *lactis* and endogenous bifidobacteria contributed to the reduction in VFA that appeared when inulin was ingested in addition to GCL2505. In addition, parameters related to lipids such as TG, TC, LDL-C and HDL-C showed no significant correlation with total bifidobacteria, endogenous bifidobacteria and *B. animalis* subsp. *lactis*. In some lactic acid bacteria, effects on blood lipids were also reported for pathways that were not mediated by SCFAs [57]. It is possible that the changes in the present parameters may have been caused by a pathway that is not mediated by SCFAs derived from bifidobacteria. This study quantified bifidobacteria but did not investigate other intestinal bacteria. Therefore, further research is needed to understand how intestinal microbiota other than bifidobacteria change and affect body composition and blood parameters.

Accumulation of visceral fat induces chronic inflammation. Recent studies have reported that intake of GCL2505 and inulin suppresses chronic inflammation, thereby improving cognitive and vascular function [58,59]. Further research is needed to determine the mechanism by which the intake of GCL2505 and inulin suppresses chronic inflammation by suppressing visceral fat accumulation.

## 5. Conclusions

In conclusion, the results suggest that the combined intake of GCL2505 and inulin suppresses the accumulation of visceral fat more compared with the intake of GCL2505 alone. Visceral adipose tissue has endocrine functions and it secretes a variety of bioactive substances, including adipocytokines, which affect the risk of developing metabolic abnormalities. In terms of preventing the development of metabolic abnormalities, reducing visceral fat is relatively more important than weight or body fat. It is thought that the combined intake of GCL2505 and inulin, which are functional food components that can be easily applied to daily life, may help to prevent the development of metabolic abnormalities in overweight adults.

## Figures and Tables

**Table 1:** Nutritional details of the test drinks.

Parameter	Placebo Group	Active Group
Energy, kcal/100 g	47.0	52.0
Moisture, g/100 g	87.0	84.8
Protein, g/100 g	2.8	2.8
Fat, g/100 g	0.1	0.1
Carbohydrate, g/100 g	9.0	11.2
Ash, g/100 g	1.1	1.1

The active drink contained 2.0 g inulin and 1.0 × 10^10^ colony-forming units GCL2505.

**Table 2:** Baseline characteristics of the participants (placebo group: *n* = 60; active group: *n* = 60).

	Characteristic	Placebo Group	Active Group	*p*-Value
Clinical findings	Age, years	50.6 (8.0)	50.6 (8.4)	0.973
	Height, cm	165.9 (8.2)	167.8 (8.5)	0.221
	Body weight, kg	73.6 (7.9)	73.8 (8.2)	0.873
	Body mass index, kg/m^2^	26.7 (1.5)	26.2 (2.0)	0.129
	Waist circumference, cm	92.5 (5.1)	92.4 (6.2)	0.902
	Hip circumference, cm	98.0 (3.6)	98.2 (4.5)	0.840
	Waist-to-hip ratio	0.9 (0.04)	0.9 (0.04)	0.720
	Visceral fat area, cm^2^	124.9 (31.0)	124.0 (31.3)	0.864
	Subcutaneous fat area, cm^2^	206.0 (58.1)	207.7 (65.2)	0.881
	Total fat area, cm^2^	330.9 (61.6)	331.7 (69.8)	0.953
	Systolic blood pressure, mmHg	127.7 (11.2)	132.4 (15.1)	0.055
	Diastolic blood pressure, mmHg	79.0 (10.6)	82.3 (11.7)	0.108
	Heartbeat, bpm	77.5 (10.5)	78.4 (10.8)	0.644
Laboratory findings	White blood cell count, /µL	6185.0 (1449)	6076.7 (1077)	0.643
	Red blood cell count, ×10^4^/µL	495.8 (45.4)	489.0 (43.8)	0.405
	Hemoglobin, g/dL	15.0 (1.3)	14.7 (1.2)	0.214
	Hematocrit, %	47.2 (3.6)	46.6 (3.2)	0.351
	Platelet count, ×10^4^/μL	26.7 (4.5)	28.0 (6.0)	0.164
	Neutrophil ratio, %	58.3 (7.2)	57.8 (5.9)	0.675
	Lymphocyte ratio, %	32.2 (6.8)	32.6 (5.7)	0.678
	Monocyte ratio, %	5.6 (1.3)	5.3 (1.0)	0.193
	Eosinophil ratio, %	3.2 (2.0)	3.4 (2.7)	0.652
	Basophil ratio, %	0.7 (0.3)	0.9 (0.3)	0.047
	Total serum protein, g/dL	7.2 (0.4)	7.3 (0.3)	0.237
	Albumin, g/dL	4.4 (0.3)	4.5 (0.3)	0.120
	Aspartate aminotransferase, U/L	24.8 (10.8)	23.5 (6.7)	0.430
	Alanine aminotransferase, U/L	30.4 (21.2)	26.6 (17.1)	0.280
	Lactate dehydrogenase, U/L	183.0 (29.6)	188.9 (32.7)	0.308
	Total bilirubin, mg/dL	0.8 (0.2)	0.9 (0.3)	0.228
	Alkaline phosphatase, U/L	75.7 (19.9)	76.8 (17.3)	0.758
Laboratory findings	γ-Glutamyl transpeptidase, U/L	40.4 (31.3)	44.7 (36.6)	0.485
	Blood urea nitrogen, mg/dL	13.3 (2.8)	13.8 (3.2)	0.356
	Creatinine, mg/dL	0.8 (0.15)	0.8 (0.16)	0.894
	Uric acid, mg/dL	5.8 (1.3)	5.8 (1.3)	0.746
	Sodium (Na), mEq/L	141.3 (1.7)	141.4 (1.4)	0.726
	Chlorine (Cl), mEq/L	104.1 (2.2)	103.9 (1.7)	0.645
	Potassium (K), mEq/L	4.2 (0.3)	4.2 (0.2)	0.145
	Calcium (Ca), mg/dL	9.5 (0.3)	9.5 (0.3)	0.294
	Total cholesterol, mg/dL	218.0 (30.2)	217.8 (31.9)	0.967
	LDL cholesterol, mg/dL	140.0 (27.5)	137.9 (30.0)	0.694
	HDL cholesterol, mg/dL	55.3 (12.4)	58.6 (14.7)	0.185
	Triglycerides, mg/dL	131.6 (65.8)	121.4 (49.7)	0.338
	Glucose, mg/dL	89.8 (9.2)	89.1 (9.6)	0.677
	HbA1c (NGSP), %	5.5 (0.3)	5.5 (0.3)	0.926
	Free fatty acid, mEq/L	0.4 (0.2)	0.5 (0.2)	0.077
	Urine pH	6.2 (0.5)	6.2 (0.6)	0.560
	Urine specific gravity	1.0 (0.007)	1.0 (0.007)	0.491
	Compliance rate of the test sample, % *	99.80 (0.50)	99.88(0.42)	0.349

All data are presented as the mean (standard deviation). Comparisons of value between placebo and active groups were tested by analysis of variance. HDL, high-density lipoprotein; LDL, low-density lipoprotein. * The compliance rate of test sample intake is shown excluding participants who dropped out.

**Table 3:** Dietary composition during the treatment period.

Parameter		0 Weeks	4 Weeks	8 Weeks	12 Weeks
Energy, kcal	Active	1820.9 (385.4)	1816.5 (428.4)	1792.3 (399.5)	1704.8 (413.2) *
	Placebo	1925.3 (360.3)	1929.8 (411.4)	1880.7 (414.8)	1820.1 (366.6) *
Protein, g	Active	68.7 (15.7)	67.3 (17.8)	66.0 (16.2)	64.9 (17.5) *
	Placebo	72.5 (15.3)	72.1 (15.4)	69.7 (19.0)	68.6 (14.9) *
Fat, g	Active	63.8 (17.9)	61.4 (19.2)	60.7 (20.0)	55.9 (19.1) *^#^
	Placebo	67.9 (20.0)	66.9 (20.4)	65.3 (20.6)	64.1 (18.7)
Carbohydrate, g	Active	229.7 (53.0)	236.2 (57.4)	233.3 (52.8)	222.6 (54.8)
	Placebo	243.3 (56.2)	247.1 (59.4)	240.4 (53.5)	230.2 (50.6) *
Dietary fiber, g	Active	10.8 (3.3)	10.7 (3.1)	10.6 (3.1)	10.1 (3.2)
	Placebo	10.9 (2.9)	11.0 (3.0)	10.4 (3.0)	9.9 (2.7) *

All data are presented as the mean (standard deviation). * *p* < 0.05 compared with week 0, paired *t*-test. ^#^ *p* < 0.05 compared with placebo, unpaired *t*-test.

**Table 4:** Changes in abdominal fat area by CT scan during the treatment period.

Parameter		0 Weeks	8 Weeks	12 Weeks	Time × Group ^†^
Visceral fat area, cm^2^	Active	124.0 (4.1)	111.1 (4.2) *	106.8 (3.6) *	<0.0001
	Placebo	119.8 (3.9)	117.5 (4.9)	114.9 (4.0)	
Subcutaneous fat area, cm^2^	Active	206.9 (8.3)	207.2 (8.3)	206.7 (8.6)	0.379
	Placebo	211.9 (8.8)	212.2 (8.6)	215.8 (9.4)	
Total fat area, cm^2^	Active	331.0 (9.3)	318.4 (9.9) *	313.5 (9.5) *	0.001
	Placebo	331.7 (9.1)	329.7 (10.2)	330.7 (10.3)	

All data are presented as the mean (standard error). * *p* < 0.05 compared with week 0, paired *t*-test. ^†^ *p*-value represented as a group-by-time interaction effect by two-factor repeated-measures analysis of variance.

**Table 5:** Changes in anthropometric parameters during the treatment period.

Parameter		0 Weeks	4 Weeks	8 Weeks	12 Weeks
Body weight, kg	Active	74.9 (1.1)	74.8 (1.1)	74.7 (1.1)	74.6 (1.1) *
	Placebo	74.8 (1.1)	74.8 (1.1)	74.6 (1.1)	74.3 (1.1)
Body mass index, kg/m^2^	Active	26.5 (0.3)	26.4 (0.3)	26.4 (0.3)	26.3 (0.3) *
	Placebo	27.0 (0.2)	27.0 (0.2)	26.9 (0.2)	26.9 (0.2)
Waist-to-hip ratio	Active	0.9 (0.005)	0.9 (0.005)	0.9 (0.005)	0.9 (0.005)
	Placebo	0.9 (0.006)	0.9 (0.005)	0.9 (0.005)	0.9 (0.005)

All data are presented as the mean (standard error). * *p* < 0.05 compared with week 0, paired *t*-test.

**Table 6:** Changes in plasma biochemistry parameters during the treatment period.

Parameter		0 Weeks	4 Weeks	8 Weeks	12 Weeks
Total cholesterol, mg/dL	Active	219.4 (4.2)	217.6 (4.1)	211.6 (4.2) *	217.5 (4.6)
	Placebo	219.2 (4.2)	219.0 (3.7)	216.2 (4.2)	219.4 (4.3)
LDL cholesterol, mg/dL	Active	139.9 (4.2)	136.3 (3.8)	135.3 (3.9) *	136.5 (4.3)
	Placebo	136.3 (3.8)	139.0 (3.4)	138.7 (3.7)	138.1 (4.0)
HDL cholesterol, mg/dL	Active	57.9 (2.1)	59.0 (1.9)	55.8 (1.7) *	56.2 (1.7)
	Placebo	55.5 (1.7)	54.6 (1.8)	54.2 (1.9)	54.5 (1.8)
Triglycerides, mg/dL	Active	113.6 (7.0)	116.2 (6.3) ^#^	113.6 (5.4) ^#^	118.3 (8.8)
	Placebo	130.2 (8.3)	141.7 (11.1)	138.5 (10.1)	143.1 (15.8)
Free fatty acid, mEq/L	Active	0.53 (0.03)	0.50 (0.02)	0.53 (0.02)	0.61 (0.03) *
	Placebo	0.48 (0.03)	0.50 (0.02)	0.48 (0.02)	0.55 (0.03) *

All data are presented as the mean (standard error). HDL, high-density lipoprotein; LDL, low-density lipoprotein. * *p* < 0.05 compared with week 0, paired *t*-test. ^#^ *p* < 0.05 compared with placebo, unpaired *t*-test.

**Table 7:** Changes in the number of fecal bifidobacterial during the treatment period.

Bifidobacteria		0 Weeks	8 Weeks	12 Weeks
Total bifidobacteria	Active	9.62 (0.14)	10.35 (0.08) *^#^	10.41 (0.07) *^#^
	Placebo	9.48 (0.17)	9.60 (0.17)	9.61 (0.17)
*B. animalis* subsp. *lactis*	Active	5.70 (0.15)	9.67 (0.09) *^#^	9.77 (0.08) *^#^
	Placebo	5.70 (0.14)	5.72 (0.15)	5.50 (0.10)
*B. longum* subsp. *longum*	Active	8.62 (0.20)	8.86 (0.23) *	8.96 (0.23) *
	Placebo	8.26 (0.23)	8.35 (0.24)	8.37 (0.23)
*B. adolescentis* group	Active	7.81 (0.28)	8.14 (0.31) *	8.18 (0.31) *
	Placebo	7.73 (0.29)	8.09 (0.29)	8.19 (0.30)
*B. catenulatum* and *B. pseudocatenulatum*	Active	8.22 (0.25)	8.40 (0.27) *^#^	8.37 (0.27) *^#^
	Placebo	7.89 (0.26)	7.87 (0.26)	7.90 (0.25)
*B. breve*	Active	5.66 (0.11)	5.77 (0.12)	5.74 (0.12)
	Placebo	5.62 (0.11)	5.62 (0.11)	5.68 (0.12)
*B. bifidum*	Active	6.02 (0.20)	6.08 (0.20)	6.14 (0.21)
	Placebo	5.89 (0.18)	5.98 (0.19)	5.98 (0.19)
*B. longum* subsp. *infantis*	Active	5.53 (0.09)	5.59 (0.11)	5.57 (0.10)
	Placebo	5.51 (0.09)	5.51 (0.10)	5.52 (0.09)
*B. dentium*	Active	5.64 (0.11)	5.66 (0.10)	5.63 (0.10)
	Placebo	5.63 (0.09)	5.71 (0.10)	5.64 (0.09)
*B. angulatum*	Active	n.d.	n.d.	n.d.
	Placebo	n.d.	n.d.	n.d.
Endogenous bifidobacteria	Active	9.60 (0.14)	9.89 (0.16) *	9.92 (0.16) *
	Placebo	9.45 (0.17)	9.57 (0.17)	9.61 (0.17)

All data are presented as the mean (standard error) of common logarithms of the number of bacteria per 1 g feces. The detection limit of quantitative PCR was 2.0 × 10^5^ cells per gram of feces. n.d., not detected. * *p* < 0.05 compared with placebo, unpaired *t*-test with Benjamini–Hochberg procedure. ^#^ *p* < 0.05 compared with week 0, paired *t*-test with Benjamini–Hochberg procedure.

**Table 8:** Association of changes over time in body composition and metabolic parameters with changes in bifidobacteria.

Object Variable	Ratio of Change from Week 0 to 12	Explanatory Variable	Change from Week 0 to 12, %	*p*-Value
Total bifidobacteria	33.46 (12.51)	BMI	−0.5 (0.2)	0.010
		VFA	−7.2 (1.3)	0.012
Endogenous bifidobacteria	27,875.92 (5977.64)	BMI	−0.5 (0.2)	0.025
*B. animalis* subsp. *lactis*	8.07 (2.63)	BMI	−0.5 (0.2)	0.025

All data are presented as the mean (standard error). Data were generated by applying analysis of variance to a mixed linear model. BMI, body mass index; VFA, visceral fat area.

## Data Availability

Datasets generated during the current study and/or analyzed during the current study are available from the responsible author upon reasonable request.

## Supplementary Materials

The following supporting information can be downloaded at: https://www.mdpi.com/article/10.3390/nu15245025/s1, Table S1: CONSORT 2010 checklist; Table S2: Changes in waist and hip circumference during the treatment period.
