# Supplementary material for: Effect of Continuous Ingestion of Bifidobacteria and Inulin on Reducing Body Fat: A Randomized, Double-Blind, Placebo-Controlled, Parallel-Group Comparison Study
Source: Nutrients. 2023 Dec 7;15(24):5025. doi: 10.3390/nu15245025 (PMC10745352; doi:10.3390/nu15245025)
Supplement: Supplementary file 1 [file nutrients-15-05025-s001.zip › Supplementary Table S2.pdf]

Table S2. Changes in waist and hip circumference during the treatment period.

Parameter		0 weeks	4 weeks	8 weeks	12 weeks
Waist circumference, cm	Active	92.5 (0.8)	92.5 (0.8)	92.2 (0.8)	92.0 (0.8) *
	Placebo	92.5 (0.7)	92.6 (0.6)	92.0 (0.7) *	92.0 (0.8)
Hip circumference, cm	Active	98.2 (0.6)	98.3 (0.6)	97.9 (0.6) *	97.9 (0.6)
	Placebo	98.4 (0.5)	98.4 (0.5)	98.0 (0.5) *	98.1 (0.5)

All data are presented as the mean (standard error).

* $p < 0.05$ compared with week 0, paired *t*-test.
